# Models solely using claims-based administrative data are poor predictors of rheumatoid arthritis disease activity

**DOI:** 10.1186/s13075-017-1294-0

**Published:** 2017-05-08

**Authors:** Brian C. Sauer, Chia-Chen Teng, Neil A. Accortt, Zachary Burningham, David Collier, Mona Trivedi, Grant W. Cannon

**Affiliations:** 1 Salt Lake City Veterans Affairs Medical Center, Health Services Research and Development (IDEAS) Center and University of Utah Division of Epidemiology, Salt Lake City, UT USA; 2 Amgen, Thousand Oaks, CA USA; 3 Salt Lake City Veterans Affairs Medical Center, Health Services Research and Development (IDEAS) Center and University of Utah Division of Rheumatology, Salt Lake City, UT USA; 4 Salt Lake IDEAS Center, VA; Salt Lake City Health Care System, 500 Foothill Drive Bldg. 182, Salt Lake City, UT 84148-0001 USA

**Keywords:** Disease activity, Statistical methods, Rheumatoid arthritis

## Abstract

**Background:**

This study developed and validated a claims-based statistical model to predict rheumatoid arthritis (RA) disease activity, measured by the 28-joint count Disease Activity Score (DAS28).

**Method:**

Veterans enrolled in the Veterans Affairs Rheumatoid Arthritis (VARA) registry with one year of data available for review before being assessed by the DAS28, were studied. Three models were developed based on initial selection of variables for analyses. The first model was based on clinically defined variables, the second leveraged grouping systems for high dimensional data and the third approach prescreened all possible predictors based on a significant bivariate association with the DAS28. The least absolute shrinkage and selection operator (LASSO) with fivefold cross-validation was used for variable selection and model development. Models were also compared for patients with <5 years to those ≥5 years of RA disease. Classification accuracy was examined for remission (DAS28 < 2.6) and for low (2.6–3.1), moderate (3.2–5.1) and high (>5.1) activity.

**Results:**

There were 1582 Veterans who fulfilled inclusion criteria. The adjusted *r*-square for the three models tested ranged from 0.221 to 0.223. The models performed slightly better for patients with <5 years of RA disease than for patients with ≥5 years of RA disease. Correct classification of DAS28 categories ranged from 39.9% to 40.5% for the three models.

**Conclusion:**

The multiple models tested showed weak overall predictive accuracy in measuring DAS28. The models performed poorly at predicting patients with remission and high disease activity. Future research should investigate components of disease activity measures directly from medical records and incorporate additional laboratory and other clinical data.

## Background

The estimated prevalence of rheumatoid arthritis (RA) among US adults is approximately 0.6% (1.5 million people age ≥18 years) [1, 2]. Administrative claims databases continue to serve as one of the largest sources of data to study RA treatment and patient outcomes. Nevertheless, the utility of these studies is limited as disease activity is not directly captured in claims databases and there is no method to directly measure disease activity in relation to treatment modification and patient outcomes that are captured in claims data. Researchers have attempted to overcome this limitation by developing claims-based indexes of disease severity. Studies have typically used a Delphi approach to identify variables thought to be associated with disease activity and tested correlation between their approach and clinical information obtained from the electronic health record [3] or existing measures of RA disease activity [4]. A recent study by Desai et al. (2015) [5] attempted to validate the Claims-based Index for Rheumatoid Arthritis Severity (CIRAS) developed by Ting et al. [3], who used the Delphi approach, against the C-reactive-protein-based 28-joint Disease Activity Score (DAS28-CRP) in a relatively small population (n = 315) of patients enrolled in the Brigham Women’s Hospital Rheumatoid Arthritis Sequential Study (BRASS) and Medicare [5]. Unfortunately, they found the correlation between CIRAS and DAS28-CRP was poor and attempts to improve the performance by adding additional claims-derived variables also performed poorly (*R* ^2^ = 0.23).

These findings highlight the need to develop an algorithm to measure disease activity/severity in observational studies, as claims data are regularly used to evaluate disease progression and response to RA therapies. The ability to measure disease activity is also important in understanding how clinicians make therapeutic decisions, determining whether patients who may benefit from more aggressive treatment are escalating treatment, and providing real-world evidence of adoption of treatment guidelines.

The goal of this study was to develop and validate claims-based statistical models to predict disease activity measured by DAS28 in a larger population of Veteran patients enrolled in the Veterans Affairs Rheumatoid Arthritis (VARA) registry.

## Methods

### Design and data

A cohort study design was used on historical data available in the VARA registry and the Veterans Administration (VA) Informatics and Computing Infrastructure (VINCI), which houses the VA Corporate Data Warehouse (CDW) data available for research. This study used VARA and VINCI data from 1 January 2006 until 31 December 2014 to predict disease activity using data typically available in administrative claims databases. The gold standard clinical DAS28 was measured in Veteran patients enrolled in the VARA registry as part of routine care.

The VARA registry is a prospective, multicenter, observational registry involving 11 VA medical centers (Birmingham, AL; Brooklyn, NY; Dallas, TX; Denver, CO; Jackson, MS; Iowa City, IA; Little Rock, AR; Omaha, NE; Portland, OR; Salt Lake City, UT; and Washington, DC). Clinical disease activity measures (i.e., DAS28 and duration of disease) were obtained from the VARA registry, which has been described elsewhere [6, 7].

All administrative claims data were obtained from VINCI [8], which contains both CDW and Managerial Cost Accounting (MCA) system data. VINCI is a research and development environment jointly funded by Health Services Research & Development Service (HSR&D) and the Office of Information and Technology (OIT). VINCI contains rich clinical data in addition to administrative claims data. As the focus of this analysis was on developing a claims-based predictor of the DAS28 we limited our predictor variables to those available in patient tables, inpatient and outpatient procedure, diagnosis and pharmacy data domains.

### Study population

Veteran patients had to be enrolled in the VARA registry to be included in the study, and had to be 18+ years of age, have a DAS28, and have at least 365 days of enrollment in the VA health care system prior to the DAS28 measurement. To ensure Veteran patients were actively using the system they were required to have had at least two encounters with the system during the 365-day baseline period. The first encounter with a DAS28 measurement that fulfilled other inclusion and exclusion criteria was eligible as the index date for training and testing the prediction model.

Veteran patients were excluded if they met any of the following criteria because their RA medications and treatment may be modified to treat cancer, transplant or other autoimmune disorders:

1. Diagnosed with any active cancer (Healthcare Cost and Utilization Project (HCUP) Single-level Clinical Classification System (CCS) [9] Diagnosis Category 11-45)
2. Undergone a transplant (HCUP CCS Procedure Category 64, 105, 176)
3. Diagnosed with other autoimmune disorders (HCUP CCS Diagnosis Category: 57, 210)

The index date was defined as the first encounter with a DAS28 after 1 January 2006 and before 31 December 2014 that fulfilled inclusion and exclusion criteria. The baseline period was defined as the 365-day observational period prior to the index date, which is the time we allowed comorbidities to accrue and contribute to the prediction of patient-level DAS.

### Study variables

#### Dependent variables

The dependent variable was the continuous DAS28, which was collected at enrollment in the VARA registry and routinely thereafter. The DAS28 is a widely used disease activity assessment tool that affects treatment decisions by rheumatologists in daily clinical practice [10]. The DAS28 is a statistically derived composite index that takes into account the patient’s number of swollen joints, number of tender joints, erythrocyte sedimentation rate (ESR), and general health using patient global assessment [11].

Our secondary endpoint was to determine if the predicted DAS28 could be correctly categorized to reflect typical clinical classification of disease, which includes remission (<2.6), low (2.6–3.1), moderate (3.2–5.1), and high (>5.1) disease activity.

#### Predictor variables

Potential predictor variables for model development were established using three distinct but complementary approaches. The first approach identified a list of potential predictors from literature review and discussion with clinical domain experts (GWC, DC, and MT). The second approach utilized hierarchical grouping software to generate drug categories, medical conditions and procedures at a feasible level for model development - described as the “all predictor” model. The third approach started with all possible predictors (i.e., *a priori* clinically defined variables and potential predictors based on hierarchical grouping systems) then used a prescreening approach that preselected variables exhibiting significant correlation with the DAS28 (*p* value <0.05). An automatic variables selection technique, described in the “Statistical approach” section, was then used to identify final sets of predictor variables for the three approaches used to identify the initial pool of potential predictor variables.

Table 4 in Appendix 1 lists the *a priori* clinically defined potential predictors used for model development. It is important to keep in mind that we restricted potential predictors to information available in administrative claims databases. For example, we recorded when specific laboratory measurements occurred but did not attempt to make use of the laboratory results. The potential baseline predictors involved patient demographics, whether specific laboratory tests or radiographs were performed, counts of clinic visits for primary, rheumatology, orthopedic, physical rehabilitation, occupational therapy, and emergency care. Hospital admissions and the number of unique drug classes served as proxies for healthcare utilization. Administrative codes were used to identify surgical procedures, hand surgery, orthopedic surgery and joint injections. Surgical procedures were identified using the Healthcare Cost and Utilization Project (HCUP) Surgical Flag Software [12, 13]. We measured specific comorbidities and implemented the Rheumatic Disease Comorbidity Index (RDCI) to account for comorbidities [14]. Procedure codes were also used to measure the use of assistive devices, such as a cane, crutch, or wheelchair.

Careful attempts were made to accurately measure disease modifying anti-rheumatic drug (DMARD) use during the baseline period and up to 14 days following the index date to reflect treatment changes in response to the DAS28. Three variables were produced for each generic ingredient and by therapeutic classes to represent: (1) days since start of therapy from the beginning of the baseline period, (2) days since ending a course of therapy from the index date (e.g., a value of 25 means the drug was discontinued 25 days before the index date), and (3) the proportion of days covered during the baseline year. Initiation and discontinuation of these therapies was also tracked during the 14 days post index date to account for the potential association between treatment modification and changes in disease activity.

In addition to the *a priori* defined potential predictors of disease activity, we leveraged existing grouping software to organize procedures, diagnoses and pharmacy claims into levels of aggregation that support prediction of RA disease activity. Specifically, we used counts of all single-level HCUP Clinical Classification System (CCS) condition and procedure groups [9]. We also used counts of all VA drug class codes (e.g., CV200: calcium channel blocker, CV300: antiarrhythmic, etc.) to account for pharmaceutical exposures, dispensed vitamins and dispensed prosthetics/supplies/devices during the baseline period (http://www.pbm.va.gov/nationalformulary.asp. - VA National Formulary by Class April 2016 Excel Spreadsheet). Dichotomous variables were required to have >1% prevalence in the population to be included in the statistical models.

### Statistical approach

Least absolute shrinkage and selection operator (LASSO) is a widely used regularization technique used for developing high-dimensional prediction models without high variance [15]. LASSO selection arises from a form of ordinary least squares regression where the sum of the absolute value of the regression coefficients is constrained to be smaller than a specified parameter. Let *X* = (*x*_1_, *x*_2_, *x*_3_…*x*_*m*_) denote the matrix of predictor variables (e.g., Table 1 with 250+ predictors) and let *y* denote the DAS28, (i.e., the response variable), where the *x*_*i*_ *s* have been centered and scaled to have unit standard deviation and mean zero and *y* has a mean of zero. For a given tuning parameter *t*, the LASSO regression coefficients minimize: ${\left\Vert \boldsymbol{y}-\boldsymbol{X}\boldsymbol{\beta } \right\Vert}^2\;\boldsymbol{subject}\;\boldsymbol{t}\boldsymbol{o}\;{\displaystyle {\sum}_{j=1}^m\left|{\boldsymbol{\mathsf{\boldsymbol{\beta}}}}_{\boldsymbol{\mathsf{j}}}\right|}\le \boldsymbol{t}$

**Table 1:** Attrition table for model development

	Inclusion/exclusion criteria	Population and encounters
1.	VARA registry patients April 1, 2015	2161 Veterans, 25,464 Encounters
2.	At least one valid DAS28	2063 Veterans, 19,064 Encounters
3.	Study period of interest (Jan 1, 2006 to Dec 31, 2014)	2002 Veterans, 17,000 Encounters
4.	Meet 365 days of enrollment criteria with ≥2 visits during baseline period	1976 Veterans with index date
5.	Remove patients with cancer	1631 Veterans
6.	Remove patients with transplant	1631 Veterans
7.	No other autoimmune disorders	1582 Veterans

*VARA* Veterans Affairs Rheumatoid Arthritis, *Das28* 28-joint disease activity score

Provided that the LASSO parameter *t* is small enough, some of the regression coefficients will be exactly zero. Therefore, the LASSO can be viewed as having a built-in variable selection technique. By increasing the LASSO parameter in discrete steps a sequence of regression coefficients is obtained, where the non-zero coefficients at each step correspond to selected parameters. The LASSO method produces a series of models, *M*_*0*_, *M*_*1*_ …..*M*_*k*_, with each model being the solution for a unique tuning parameter value. In this series, *M*_*0*_ can be thought of as the least complex model, for which the maximum penalty is imposed on the regression coefficient, and *M*_*k*_ is the most complex model, for which no penalty is imposed. The prediction error is then computed for each model and the model that yields the minimum prediction error is chosen.

Cross-validation is important in guarding against overfitting the model to the data, meaning the model is fit to random error instead of true underlying relationships among the variables. Overfitting reduces model performance when applied to an independent dataset that was not involved in the training of the model. Fivefold “external” cross-validation was applied using randomly selected folds to train and test the statistical model. Every fold was used for both training and testing, e.g., when fold 0 was the testing set then folds 1–4 were used for training and when fold 4 was the testing set then folds 0–3 were used for training. SAS GLMSELECT (SAS version 9.4 with Enterprise Guide version 6.1 (Cary, NC, USA)) was used to implement the cross-validated LASSO procedure and the model that minimizes the cross-validated external predicted sum of squares was used as the primary model selection criterion [16].

### Classification analysis

As the secondary study endpoint, we attempted to use the predicted DAS28 to correctly classify patients who were categorized into typical clinical categories of remission and low, moderate, and high disease activity using the actual DAS28.

The correct classification rate (CCR) was used to determine how well the predicted values were classified into the four clinical groups based on the clinical DAS28. CCR is the percentage of correct observations (suitability with the expected value). CCR can be calculated using the following formula:

$$ C C R\kern0.5em =\kern0.5em \frac{number\kern0.5em  of\kern0.5em  correct\kern0.5em  prediction}{number\kern0.5em  of\kern0.5em  observation}\kern0.5em  x\ 100\% $$

The higher percentage of CCR shows higher accuracy [17]. Exact binomial 95% confidence internals were computed for the CCR [18].

### Sensitivity analysis

We developed three models that varied by the initial pool of variables available for model development. As described above, the first model comprised the clinically defined variables identified from our comprehensive literature review and use of clinical domain experts. The second model comprised these same clinically defined variables, plus HCUP CCS condition and procedure codes, and Veterans Health Administration (VHA) drug class codes. The third model comprised variables from the second model; however, only those variables statistically associated with the DAS28 were included (prescreening). Additional sensitivity analysis was applied on duration of RA disease. Models were compared for RA patients with <5 years of disease and ≥5 years of disease.

## Results

### Population

During the observation period from 1 January 2006 to 31 December 2014 there were 1582 VARA patients meeting all inclusion criteria for the model development phase. Study attrition is presented in Table 1. The average age at index for the population was 63 years (standard deviation (SD): 11) and 90% (95% CI: 88–91%) of the population were male. The DAS28 was normally distributed with an average DAS28 of 3.8 (SD: 1.54) and average Rheumatoid Disease Comorbidity Index (RDCI) of 2.2 (SD: 1.6).

### Model development and testing

The clinical experts identified 253 *a priori* claims-based variables representing clinical concepts thought to be associated with the DAS28. After applying the restriction that variables must be present in >1% of the population we ended up with 175 potential predictor variables. After model development with the LASSO regularized regression, the final model contained 32 variables and the adjusted *R*-square was 0.221 (Table 2). The “all predictors” and prescreening models started with different initial potential predictor variables but produced similar cross-validated adjusted *R*-square values, 0.221 and 0.223, respectively.

**Fig. 1:**
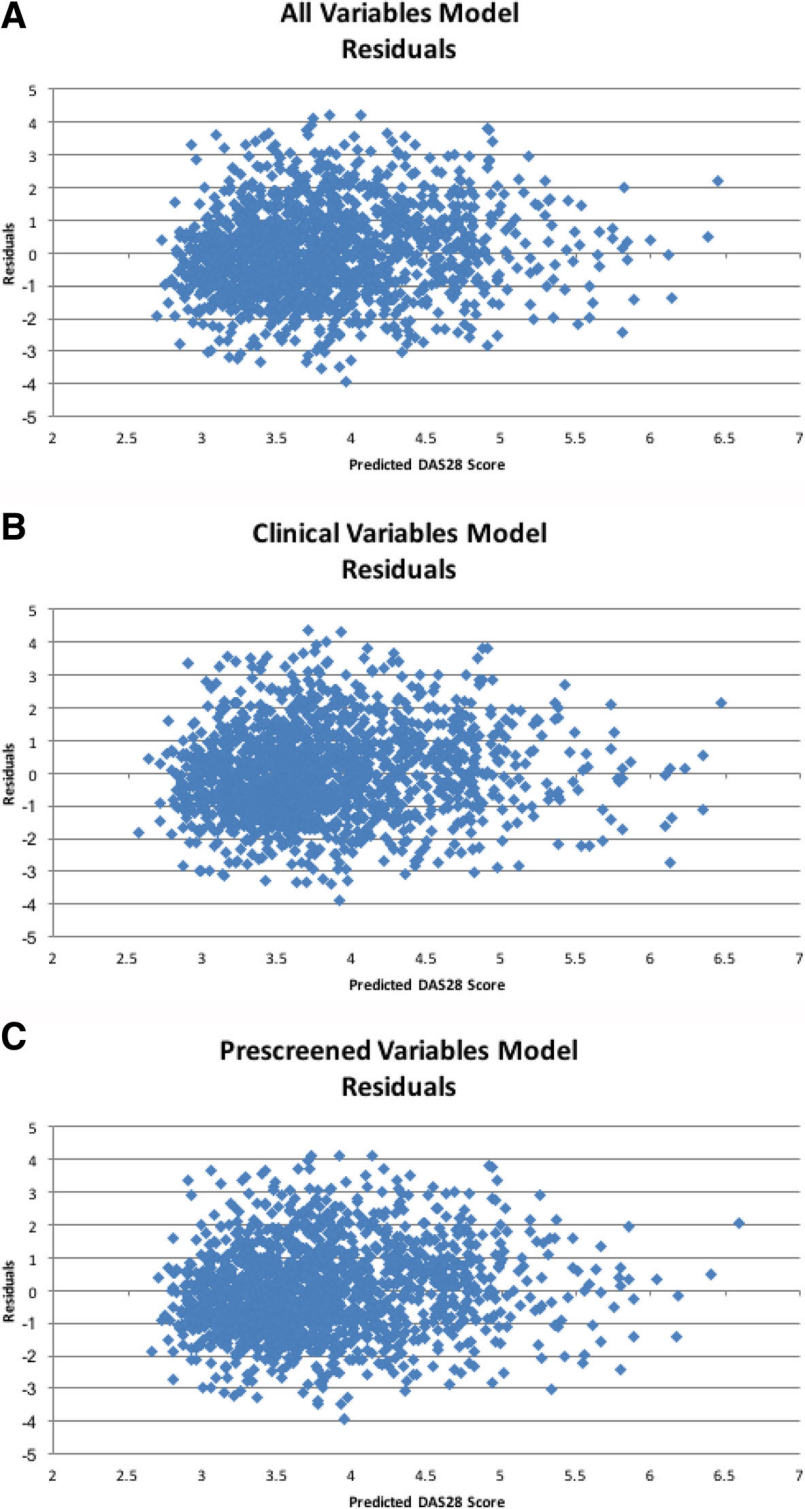
Predicted 28-joint disease activity score (DAS28) vs true DAS28 for test data. **a** Clinical predictors. **b** All predictors. **c** Pre-screened variables

Review of the scatter plots presented in Fig. 1a-c show that the predicted DAS28 did not contain the same range of values as the true DAS28. The predicted DAS28 overestimated those with low DAS28 and underestimated the predicted value for Veteran patients with high DAS28.

**Table 2:** Model validation by initial set of predictor variables

Variable sets	All clinical predictors	All predictors	Prescreening
Potential predictors	253	1275	279
Potential predictors with ≥1% prevalence (i.e., predictors put into model)	175	567	230
Predictors in final model	32	45	46
Fivefold cross-validated *R*-square (test)	0.237	0.243	0.246
Fivefold cross-validated Adj *R*-square (test)	0.221	0.221	0.223
<5 Years of RA disease Adj *R*-square	0.218	0.190	0.196
≥5 years of RA disease Adj *R*-square	0.186	0.185	0.185

*RA* rheumatoid arthritis, *Adj* adjusted

### Model-retained predictor variables

Variables selected and their standardized estimates for each model are presented in Appendix 2: Tables 5-7. Variables are ordered by the magnitude of the standardized estimate. Healthcare utilization (total visit count, primary care visits and occupational health visits) were relatively strong predictors of higher DAS28. Starting a new DMARD (biologic or non-biologic) 14 days after the DAS28 measurement was also consistently associated with higher DAS28. Higher baseline proportion of days covered (PDC) for DMARDs during the prior year was associated with lower DAS28. Measures indicating higher comorbidity, such as the RDCI and number of distinct VA drug classes were associated with higher DAS28. In the two models that used grouping software, we found analgesics, antidepressants and other agents acting on the central nervous system to be associated with higher DAS28. Please review Appendix 2 for a complete list of variables retained in each model.

### Secondary analysis: correct classification of disease activity categories

The overall CCR for correctly classifying patients with the predicted DAS28 into clinical categories based on the true DAS28 ranged from 39.9% to 40.5% (Table 3). The true positive rate (TPR), which essentially represents measurement sensitivity, was fairly low in the group classified as having high disease activity by the predicted DAS28 and ranged from 9.9% to 10.8% (Table 3), while the positive predicted value (PPV) ranged from 57.9% to 63.0%. The TPR for those classified as having moderate disease ranged from 84.6% to 87.6%, while the PPV ranged from 42.5% to 43.0%. The TPR for low disease acitivity ranged from 17.9% to 20.2%, while the PPV ranged from 21.0% to 21.6%. None of the prediction models developed and tested accurately classified patients with remission. The TPR ranged from 0.0% to 0.3%, while the PPV ranged from 100% to 0%. Attempts to statistically define the cut points for the predicted DAS28 to optimize correct classification did not meaningfully improve the CCR. The optimized CCR only increased by approximately 3% (not presented).

**Table 3:** Classification accuracy by disease activity category

			All clinical predictors	All predictors	Prescreening
4 Categories	High (>5.1)	TPR	10.5%	9.9%	10.8%
		PPV	57.9%	62.0%	63.0%
	Moderate (3.2–5.1)	TPR	84.6%	87.6%	86.9%
		PPV	42.5%	43.0%	43.0%
	Low (2.6–3.1)	TPR	20.2%	17.9%	18.3%
		PPV	21.6%	21.2%	21.0%
	Remission (<2.6)	TPR	0.3%	0.0%	0.0%
		PPV	100.0%	0.0%	0.0%
	CCR (95% CI)		39.9% (37.5–42.3%)	40.5% (38.1–43.0%)	40.5% (38.1–43.0%)

*CCR* correct classification rate, *TPR* true positive rate, *PPV* positive predicted value

## Discussion

This study developed and internally validated statistical models to predict RA disease activity using data available in administrative claims data but was not successful in establishing a high level of predictive value. The relatively low adjusted *R*-square value and the inability to accurately classify patients into categories of disease activity using the predicted DAS28, draw into question the ability of a claims-based predictor to successfully represent patient disease activity in RA.

The sensitivity analysis focused on development of multiple models based on the initial pool of variables and applying these models to subsets of patients with <5 years of RA disease and ≥5 years of disease. This cut point was chosen because it has been used in other studies (e.g., TICORA 2004 study) [19] to represent early disease and because the data did not support comparisons with earlier cut-points.

The initial pool of predictors did not impact predictive accuracy, as the three models had similar adjusted *R*-square values. The models performed slightly better for patients with <5 year of disease. Similar results were found for patients with <2 years of disease (not presented). Duration of RA disease was recorded at VARA enrollment and would not be available in claims data, but could be approximated based on duration of enrollment and indicators of disease in claims-based data.

Variables retained in the three models were intuitive and interpretable. Variables indicating increased healthcare utilization, comorbidities, the use of agents that act on the central nervous system, and new DMARD prescriptions after the DAS28 measurement were associated with higher DAS28. Variables indicating longer exposure and a higher proportion of days covered by DMARDs in the baseline period were associated with lower DAS28. Even though many variables were associated with DAS28 the models poorly explained the variation in DAS28.

The classification accuracy of predicted DAS28 was evaluated by categorizing the true DAS28 and predicted DAS28 into remission (<2.6), low disease activity (2.6–3.1), moderate disease activity (3.2–5.1), and high disease activity (>5.1). The overall classification accuracy across the three models was modest. The models were poor predictors of remission and high levels of disease activity, which is likely due to the relatively small number of Veterans in the lower and higher end of the DAS28 scale. They performed best at classifying patients with moderate disease activity, which is the range for the majority of the population. In an additional analysis (not presented), we identified the optimal cut points of the predicted DAS28 to correctly classify patients by true DAS28 category of disease activity but were only able to increase classification accuracy to 43% across the three models.

A recent study evaluating the accuracy of a claims-based model to predict DAS28-CRP also performed poorly and found other claims-based measures of RA disease severity were not well correlated with true DAS28-CRP. Desai et al. (2015) [5] evaluated the claims-based index for RA severity (CIRAS) developed by Ting et al. [3] using a Delphi panel and found poor correlation with the true DAS28-CRP. They added additional variables to CIRAS that included medical claims for rheumatoid lung involvement, hand surgery, tuberculin test ordered and anti-CCP test orders. Furthermore, they also added pharmacy claims for steroids, opioids, non-steroidal anti-inflammatory drugs (NSAIDs), number of non-biologic DMARDs, and number of biologic DMARDs, and found the model *R*-square value (0.23) was similar to ours, even though they had a relatively small sample of Medicare patients (n = 315). They concluded that CIRAS may not approximate RA disease in observational cohorts and its use for confounding adjustment should be carefully considered. They also concluded that claims-based algorithms developed for clinical disease activity should be rigorously tested in a population with actual measures of disease activity to establish their generalizability before implementing in research. Our study leveraged a substantially larger population and rigorously tested our models to reduce the likelihood of overfitting the models to the data, yet had similar results.

### Strength and limitations

The primary strength of this study was the access to DAS28 in a relatively large number of patients. This is the largest study that attempted to train and validate a claims-based predictor of RA disease activity using the actual DAS28. In addition, we used statistical methods that supported high-dimensional variable selection and prevented overfitting the model to the actual data. Of note, we replicated model development and classification accuracy using the Clinical Disease Activity Index (CDAI) and found similar adjusted *R*-square values and classification accuracy (results not presented).

One weakness of this study is that the population studied may not represent average RA patients. Veterans tend to be older and more likely male than the general population. Another weakness of this study was the reliance on claims-based predictors. Even though observational comparative effectiveness and safety studies often rely on commercial and Federal claims databases there is a new paradigm for population health analytics that involves the integration of the electronic health record with claims data. Accurate prediction models of disease activity will likely require integration of clinical data, such as laboratory test results and information captured in structured electronic health data or in medical notes. The use of standardized templates would be required to extract components of disease activity scores, such as the number of swollen or tender joints, and used in models to predict future disease activity and to better understand what features of the disease activity score influence changes in treatment patterns. Our future work aims to develop natural language processing tools to extract components of the DAS28 from templated notes within the VA and use this information along with other clinical data in the development of future RA disease activity prediction models.

## Conclusions

The prediction models developed and tested found a relatively low level of predictive accuracy, drawing into question their use for confounding adjustment or to evaluate treatment decisions in patients with predicted DAS28. Our findings are consistent with other recent studies that attempted to use claims-based data to predict RA disease activity. Future work to predict disease activity in patient populations should incorporate clinical data from the electronic health record in addition to key variables available in administrative claims data.

## Appendix 1

**Table 4:** List of *a priori* Clinically Defined Predictors and their Measurement

Measurement	Value	Variable	Table
Laboratory			
Number ESR tests	85651, 85652	CPT	CDW.OutpatProcedure
Number CRP tests	86140	CPT	CDW.OutpatProcedure
aCCP – (Y/N)	86200	CPT	CDW.OutpatProcedure
RF – (Y/N)	86431	CPT	CDW.OutpatProcedure
Number. complete blood counts	85025	CPT	CDW.OutpatProcedure
Number. chemistry panels ordered or any electrolyte	80053, 82248, 82465, 82977, 83540, 83615, 84100, 84478, 84550	CPT	CDW.OutpatProcedure
Radiology			
Radiographs (Y/N)	70010-79999	CPT	CDW.OutpatProcedure
Number radiographs	Count of 70010-79999	CPT	CDW.OutpatProcedure
Hand or foot (Y/N) (toes, fingers, wrist)	73620, 73630, 73650, 73660, 73100, 73110,73120, 73130	CPT	CDW.OutpatProcedure
C-spine (Y/N)	72040, 75052	CPT	CDW.OutpatProcedure
Other bone x-ray (Y/N)	Master file	CPT	CDW.OutpatProcedure
Clinic Visits			
Any visit	Any	stopcode	CDW.outpatVisit
Rheumatology	314	stopcode	CDW.outpatVisit
Primary care	323, 342	stopcode	CDW.outpatVisit
Orthopedic	60, 509	stopcode	CDW.outpatVisit
Physical Rehabilitation	201, 250	stopcode	CDW.outpatVisit
Occupational Therapy	21, 206, 460	stopcode	CDW.outpatVisit
Number ED visits	130, 101	stopcode	CDW.outpatVisit
Utilization			
Number Inpatient stay	Row count per person	Admission date	Inpatient.inpatient
Unique Drug class codes	Row count per person	VAdrugclasscode	CDW.Rxoutpat
Procedures			
Any surgery	10021-6990	CPT	
Any surgery	Master file	ICD9	CDW.outpatProcedure, inpatProcedure, inpatientCPT
Hand surgery	26500, 26502, 26504, 26494, 26989	CPT	CDW.Outpat.Procedure, inpatientCPT
Hand surgery	Master file^a^	ICD9	CDW.inpatientProcedure
Any orthopedic (include spine) surgery	Master file	CPT	CDW.inpatientCPT, Outpat.Procedure
Joint injections	Master file	Jcode	CDW.Outpat.Procedure, inpatientCPT
Comorbidity			
RDCI RA comorbidity score	See published paper	ICD9	CDW.outpatDiagnosis
Rheumatoid lung involvement	714.81	ICD9	CDW.outpatDiagnosis
Felty’s syndrome	714.1	ICD9	CDW.outpatDiagnosis
Sjogrens	710.2	ICD9	CDW.outpatDiagnosis
Assistive Devices			
Cane (Y/N)	Master file	CPT/ICD9 procedure	CDW.outpatProcedure, inpatProcedure
Crutch (Y/N)	Master file	CPT/ICD9 procedure	CDW.outpatProcedure, inpatProcedure
Walker (Y/N)	Master file	CPT/ICD9 procedure	CDW.outpatProcedure, inpatProcedure
Wheelchair (Y/N)	Master file	CPT/ICD9 procedure	CDW.outpatProcedure, inpatProcedure
Other Assist Device (Y/N)	Master file	CPT/ICD9 procedure	CDW.outpatProcedure, inpatProcedure
Medications dispensed			
Number NSAID	MS102	VA drug Class	CDW.Rxoutpatfill
Number Non-biologic DMARD	String search - list below	drugnamewithoutdose	CDW.Rxoutpatfill
Methotrexate (Y/N)	String search	drugnamewithoutdose	CDW.Rxoutpatfill
Leflunomide (Y/N)	String search	drugnamewithoutdose	CDW.Rxoutpatfill
Sulfasalazine (Y/N)	String search	drugnamewithoutdose	CDW.Rxoutpatfill
Hydroxychloroquine (Y/N)	String search	drugnamewithoutdose	CDW.Rxoutpatfill
Azathioprine (Y/N)	String search	drugnamewithoutdose	CDW.Rxoutpatfill
TNFi (Y/N)	String search: etanercept, adalimumab, infliximab, golimumab, certolizumab	drugnamewithoutdose	CDW.MCA, Rxoutpatfill, outpatProcedure, TIU notes
Other biologics	rituximab, abatacept, anakinra, tocilizumab,	drugnamewithoutdose	CDW.MCA, Rxoutpatfill, outpatProcedure, TIU notes
Tofacitinib (Y/N)	String search	drugnamewithoutdose	CDW.Rxoutpatfill
Glucocorticosteroids (Y/N and Dose)	HS051	VA drug Class	CDW.MCA and Rxoutpatfill
Minor DMARDs (Any Y/N)	Doxycyline, minocycline, auranofin, d-penicillamine, cyclosporine	drugnamewithoutdose	CDW.Rxoutpatfill
Smoking cessation medication (Y/N)	Chantix Nicotine substitutes	Drugnamewithoutdose	CDW.Rxoutpatfill
Processed Medication Data for DMARDs			
Days since start med	All meds	drugnamewithoutdose	CDW.Rxoutpatfill
Days since end med (90-day gap to end)	All meds	drugnamewithoutdose	CDW.Rxoutpatfill
PDC from start therapy to end of observation period	All meds	drugnamewithoutdose	CDW.Rxoutpatfill
Demographics			
Age	Age	DOB	CDW.patient
Gender	M/F	Gender	CDW.patient

^a^Master File = primary excel workbook containing all measured concepts and is available by request (brian.sauer@utah.edu)

*ESR* erythrocyte sedimentation rate, *CRP* c-reactive protein, *PDC* proportion of days covered

## Appendix 2

**Table 5:** Model Generated from Clinically Defined Predictors

Effect	Estimate	Standardized Est
Intercept	4.639526	0
Baseline Visit Count	0.001981	0.02366
Baseline RDCI Score	0.005026	0.00521
Baseline Labtest Days for Chem Panel	-0.001423	-0.0026
Baseline Primary Care Visit Count	0.005222	0.01475
Baseline Occupational Visit Count	0.010707	0.01553
Bupropion Rx in Baseline (Y/N)	0.106174	0.0135
Distinct VA Drug Class Rx	0.030526	0.13957
Joint Injection Count	0.019454	0.0097
Rheumatism, Unspecified And Fibrosis Dx Count (ICD:729)	0.008102	0.00519
Baseline Surgery Count	-0.058006	-0.0162
Days End of Prednisone	-0.000912	-0.09845
Days End of Sulfasalazine	-0.000063599	-0.00505
Baseline PDC for Etanercept	-0.152669	-0.02073
Days Start of Golimumab	-0.001374	-0.00927
Baseline PDC for Non-Biologic DMARDs	-0.708075	-0.16342
Start Methotrexate in 14 Days after Index Date (Y/N)	0.077462	0.01148
Start Leflunomide in 14 Days after Index Date (Y/N)	0.073709	0.00598
Start in TNFi Biologic DMARDs in 14 Days after Index Date (Count of distinct Rx)	0.542521	0.06171
Start in Non-Biologic DMARDs in 14 Days after Index Date (Count of distinct Rx)	0.380811	0.09612
Start in Steroid in 14 Days after Index Date (Count of distinct Rx)	0.45952	0.06632
Rheumatism, Unspecified And Fibrosis Dx (ICD:729) (Y/N)	0.003294	0.00075
Baseline Labtest for Chem Panel (Y/N)	-0.482788	-0.0294
Baseline Labtest for RF (Y/N)	0.022162	0.00714
Baseline Foot Surgery (Y/N)	0.037806	0.01158
Baseline Hand Surgery (Y/N)	0.105081	0.02942
Baseline Occupational Visit (Y/N)	0.116702	0.02344
Baseline Rheumatology Visit (Y/N)	-0.033104	-0.00143
Start in Non-Biologic DMARDs in 14 Days after Index Date (Y/N)	0.340854	0.07459
Start in DMARDs in 14 Days after Index Date (Y/N)	0.590044	0.08356
Baseline Use of Sulfasalazine (Y/N)	0.158241	0.03893
Baseline Use of Rituximab (Y/N)	0.112073	0.00872
Baseline Use of Infliximab (Y/N)	0.003816	0.00057

**Table 6:** Model Generated from All Predictors

Effect	Estimate	Standardized Est
Intercept	4.027342	0
Baseline Visit Count	0.002189	0.02615
Baseline Occupational Visit Count	0.008477	0.01229
Bupropion Rx in Baseline (Y/N)	0.020813	0.00265
Non-Opioid Analgesics Rx Count (CN103)	0.001982	0.005
Sedative/Hypnotics, Other Rx Count (CN309)	0.002925	0.00222
Tricyclic Antidepressants Rx Count (CN601)	0.001475	0.00207
Local Anesthetics, Topical Rx Count (DE700)	0.016648	0.00492
Calcium Rx Count (TN402)	-0.050907	-0.01654
Decongestants, Systemic Rx Count (RE200)	0.030669	0.00639
Platelet Aggregation Inhibitors Rx Count (BL700)	0.001719	0.00095
Distinct VA Drug Class Rx	0.017323	0.07921
Baseline Surgery Count	-0.007242	-0.00202
Days End of Prednisone	-0.000873	-0.09417
Baseline PDC for Etanercept	-0.102578	-0.01393
Days Start of Golimumab	-0.000198	-0.00133
Baseline PDC for Non-Biologic DMARDs	-0.662683	-0.15295
Start Methotrexate in 14 Days after Index Date (Y/N)	0.037588	0.00557
Start in TNFi Biologic DMARDs in 14 Days after Index Date (Count of distinct Rx)	0.446665	0.05081
Start in Non-Biologic DMARDs in 14 Days after Index Date (Count of distinct Rx)	0.352313	0.08893
Start in Steroid in 14 Days after Index Date (Count of distinct Rx)	0.173564	0.02505
Disease of Mouth Excluding Dental Dx Count in Baseline (CCS137)	0.03199	0.00289
Calculus of Urinary Tract Dx Count in Baseline (CCS160)	-0.013249	-0.00541
Other Non-Traumatic Joint Disorders Dx Count in Baseline (CCS204)	0.000382	0.00074
Other Connective Tissue Disease Dx Count in Baseline (CCS211)	0.00293	0.00464
Disease of White Blood Cells Dx Count in Baseline (CCS63)	0.044243	0.00827
Conditions Associated With Dizziness or Vertigo Dx Count in Baseline (CCS93)	0.005453	0.00187
Baseline Labtest for Chem Panel (Y/N)	-0.314001	-0.01912
Baseline Foot Surgery (Y/N)	0.006726	0.00206
Baseline Hand Surgery (Y/N)	0.106317	0.02977
Opioid Analgesics Rx (CN101) (Y/N)	0.183042	0.05861
Non-Opioid Analgesics Rx (CN103) (Y/N)	0.065908	0.02006
Tricyclic Antidepressants Rx (CN601) (Y/N)	0.080738	0.01271
Antidepressants, Other Rx (CN609) (Y/N)	0.059894	0.01824
CNS Medications, Other Rx (CN900) (Y/N)	0.047949	0.00365
Loop Diuretics Rx (CV702) (Y/N)	0.073401	0.01455
Diagnostics, Other Rx (DX900) (Y/N)	0.142061	0.03273
Insulin Rx (HS051) (Y/N)	0.002114	0.00069
Thyroid Supplements Rx (HS851) (Y/N)	0.00651	0.0013
Immune Suppressants Rx (IM600) (Y/N)	0.033029	0.0059
Calcium Rx (TN420) (Y/N)	-0.080805	-0.01165
Baseline Occupational Visit (Y/N)	0.045223	0.00908
Start in Non-Biologic DMARDs in 14 Days after Index Date (Y/N)	0.375004	0.08206
Start in DMARDs in 14 Days after Index Date (Y/N)	0.816375	0.11562
Baseline Use of Sulfasalazine (Y/N)	0.144504	0.03555
Baseline Use of Rituximab (Y/N)	0.028977	0.00226

**Table 7:** Model Generated from Pre-screened Predictors

Effect	Estimate	Standardized Est
Intercept	4.268844	0
Baseline Visit Count	0.001667	0.01991
Baseline Primary Care Visit Count	0.000924	0.00261
Baseline Occupational Visit Count	0.011031	0.016
Bupropion Rx in Baseline (Y/N)	0.052414	0.00666
Non-Opioid Analgesics Rx Count (CN103)	0.003163	0.00798
Anti-rheumatics, Other Rx Count (MS190)	-0.000129	-0.00032
Sedative/Hypnotics, Other Rx Count (CN309)	0.00711	0.0054
Tricyclic Antidepressants Rx Count (CN601)	0.002659	0.00374
Local Anesthetics, Topical Rx Count (DE700)	0.026352	0.00779
Soaps/Shampoos/Soap-Free Cleansers Rx Count (DE400)	0.012432	0.00283
Decongestants, Systemic Rx Count (RE200)	0.055989	0.01166
Distinct VA Drug Class Rx	0.014658	0.06702
Joint Injection Count	0.003921	0.00196
Days End of Prednisone	-0.000913	-0.09852
Baseline PDC for Etanercept	-0.131689	-0.01788
Days Start of Golimumab	-0.000651	-0.00439
Baseline PDC for Non-Biologic DMARDs	-0.672468	-0.1552
Start Methotrexate in 14 Days after Index Date (Y/N)	0.04705	0.00697
Start Leflunomide in 14 Days after Index Date (Y/N)	0.013983	0.00113
Start in TNFi Biologic DMARDs in 14 Days after Index Date (Count of distinct Rx)	0.471838	0.05367
Start in Non-Biologic DMARDs in 14 Days after Index Date (Count of distinct Rx)	0.364512	0.09201
Start in Steroid in 14 Days after Index Date (Count of distinct Rx)	0.175888	0.02539
Varicose Veins of Lower Extremity (CCS119)	0.020937	0.00268
Disease of Mouth Excluding Dental Dx Count in Baseline (CCS137)	0.064254	0.00581
Other Connective Tissue Disease Dx Count in Baseline (CCS211)	0.003949	0.00625
Disease of White Blood Cells Dx Count in Baseline (CCS63)	0.066725	0.01247
Conditions Associated With Dizziness or Vertigo Dx Count in Baseline (CCS93)	0.013165	0.0045
Baseline Lab test for Chem Panel (Y/N)	-0.378938	-0.02308
Baseline Lab test for RF (Y/N)	0.001583	0.00051
Baseline Foot Surgery (Y/N)	0.019935	0.00611
Baseline Hand Surgery (Y/N)	0.10473	0.02932
Opioid Analgesics Rx (Y/N)	0.190656	0.06105
Non-Opioid Analgesics Rx (CN103) (Y/N)	0.070215	0.02137
Tricyclic Antidepressants Rx (CN601) (Y/N)	0.098618	0.01553
Antidepressants, Other Rx (CN609) (Y/N)	0.064811	0.01974
CNS Medications, Other Rx (CN900) (Y/N)	0.095795	0.00729
Loop Diuretics Rx (CV702) (Y/N)	0.084218	0.01669
Diagnostics, Other Rx (DX900) (Y/N)	0.15962	0.03678
Insulin Rx (HS501) (Y/N)	0.013138	0.00212
Thyroid Supplements Rx (HS851) (Y/N)	0.02656	0.00532
Antigout Agents Rx (MS400) (Y/N)	0.003596	0.00046
Baseline Occupational Visit (Y/N)	0.052566	0.01056
Start in Non-Biologic DMARDs in 14 Days after Index Date (Y/N)	0.357809	0.0783
Start in DMARDs in 14 Days after Index Date (Y/N)	0.834045	0.11812
Baseline Use of Sulfasalazine (Y/N)	0.157077	0.03865
Baseline Use of Rituximab (Y/N)	0.08026	0.00625

## Abbreviations

BRASS: Brigham Women’s Hospital Rheumatoid Arthritis Sequential Study
CCR: Correct classification rate
CCS: Clinical Classification System
CDAI: Clinical Disease Activity Index
CDW: Corporate Data Warehouse
CIRAS: Claims-base Index for Rheumatoid Arthritis Severity
DAS28: 28-Joint Disease Activity Score
DAS28-CRP: C-reactive protein-based 28-Joint Disease Activity Score
DMARD: Disease modifying anti-rheumatic drug
ESR: Erythrocyte sedimentation rate
HCUP: Healthcare Cost and Utilization Project
HSR&D: Health Services Research & Development Service
LASSO: Least absolute shrinkage and selection operator
MCA: Managerial Cost Accounting
OIT: Office of Information and Technology
PDC: Proportion of days covered
PPV: Positive predicted value
RA: Rheumatoid arthritis
RDCI: Rheumatic Disease Comorbidity Index
SEAC: Scientific, Ethical, and Advisory Committee
TPR: True positive rate
VA: Veterans Administration
VARA: Veterans Affairs Rheumatoid Arthritis
VINCI: Veterans Administration (VA) Informatics and Computing Infrastructure
